# Multidimensional Determinants of Food and Nutritional Insecurity Among Older Adults: A Scoping Review

**DOI:** 10.3390/healthcare14101396

**Published:** 2026-05-20

**Authors:** Pedro Lima, Eliane Rezende, Carmem Piagge, Estefanía Canedo, Maria Lucia Robazzi

**Affiliations:** 1 Department of Gerontology, Health Sciences Center (CCS), Federal University of Paraíba (UFPB), João Pessoa 58051-900, Brazil; pedrohenrrique_mme@hotmail.com; 2 Department of Nutrition, Federal University of Alfenas (MG), Alfenas 37130-001, Brazil; eliane.rezende@unifal-mg.edu.br; 3 Department of Restorative Dentistry, Health Sciences Center (CCS), Federal University of Paraíba (UFPB), João Pessoa 58051-900, Brazil; carmem.piagge@academico.ufpb.br; 4 School of Nursing of Salamanca, University of Salamanca, 37008 Salamanca, Spain; 5 School of Nursing of Ribeirão Preto, University of São Paulo (USP), Ribeirão Preto 14040-901, Brazil; avrmlccr@eerp.usp.br

**Keywords:** food insecurity, nutrition, older adults, public health, scoping review, social determinants of health

## Abstract

**Background/Objectives**: Food and nutritional insecurity (FNI) is a major social determinant of health that disproportionately affects older adults, with significant implications for their health, nutrition, and well-being. In this context, this scoping review aims to map and synthesize the available scientific evidence on the main determinants of FNI among older adults, considering socioeconomic, health-related, functional, psychosocial, and structural factors. **Methods**: A scoping review was conducted in accordance with the Joanna Briggs Institute methodology and reported following the PRISMA-ScR guidelines. A comprehensive search was performed across eight databases (PubMed/MEDLINE, EMBASE, Scopus, Web of Science, CINAHL, LILACS, ProQuest, and Google Scholar), up to November 2024. Original studies addressing FNI in individuals aged ≥60 years were included. Study selection and data extraction were conducted independently by two reviewers, with disagreements resolved by consensus. **Results:** Of 5897 records identified, 15 studies met the inclusion criteria. FNI in older adults was described as a multifactorial phenomenon associated with low income, limited education, social isolation, widowhood, chronic diseases, functional limitations, depressive symptoms, and poor housing conditions. Structural determinants, including institutional racism, gaps in social protection systems, and barriers to accessing food assistance programs, were also reported. Considerable heterogeneity in measurement instruments highlights the complexity of assessing FNI in this population. **Conclusions**: Addressing FNI in older adults requires moving beyond isolated interventions toward integrated, intersectoral strategies that tackle its underlying social and structural drivers. Strengthening social protection systems, reducing access barriers, and promoting equity-oriented policies are essential to ensure adequate nutrition and support healthy and dignified aging.

## 1. Introduction

Food and nutritional insecurity (FNI) is widely recognized as a major social determinant of health, particularly among older adults, due to its strong association with adverse outcomes in health, nutritional status and overall well-being [1,2]. In a landscape of accelerated demographic transition, the rapid growth of the older population, especially in low -and middle- income countries, has increased the visibility of conditions that compromise quality of life in later stages of life, including difficulties in maintaining regular and dignified access to sufficient and adequate food [3]. Structural, economic and social inequalities tend to intensify with aging, making FNI an increasingly complex and pressing public health phenomenon.

Population aging occurs at different paces in developed and developing countries. In the latter, the growth of the older population has happened more rapidly, often without the necessary accompaniment of effective public social protection policies [3]. Older adults frequently face economic barriers, such as reduced income after retirement and increased healthcare and medication costs, alongside physical and functional limitations that hinder food preparation and access, thereby increasing their vulnerability [4]. Social isolation and the absence of support networks are also factors that exacerbate this vulnerability, compromising nutritional status and psychosocial well-being [5].

Studies indicate that FNI in older adults is a multifactorial phenomenon, influenced by a wide range of determinants, including socioeconomic conditions, health status, functional capacity, significant life events (such as widowhood or changes in housing), and psychological factors such as depression and anxiety [2,6]. These determinants interact dynamically over time, contributing to the unstable and progressive nature of FNI, which can range from mild to severe and substantially impact quality of life [7]. However, despite the growing body of literature, current evidence remains fragmented, with considerable variability in how FNI is conceptualized and measured, as well as in the relative importance attributed to its determinants across different contexts.

Moreover, inconsistencies in assessment instruments and contextual differences between populations have led to divergent findings, limiting the comparability and generalizability of existing studies. This lack of standardization poses significant challenges for accurately identifying at-risk populations and designing effective interventions. Furthermore, older adults represent a group that may have a reduced capacity to advocate for their rights, and their social vulnerability is often overlooked. In this sense, FNI should be understood not only as a public health issue but also as a matter of social justice, reflecting inequalities in access to essential resources such as adequate food [8].

Previous systematic reviews have contributed to understanding factors related to food choice [9] and food insecurity [10] in older populations; however, their scope has been limited in important ways. For instance, a systematic review focusing on food choice among independently living older adults identified a broad range of influencing factors, including physiological changes, psychosocial aspects, and personal resources, highlighting the complex interplay shaping dietary behaviors in later life [9]. Nevertheless, this body of evidence is largely based on studies conducted prior to 2015 and is often restricted to specific contexts, limiting its applicability to current demographic and socioeconomic realities.

Similarly, research examining the measurement of food insecurity has demonstrated that existing tools predominantly capture only a single dimension, most commonly food access, while failing to adequately assess other critical dimensions such as utilization, availability, and stability over time [11]. This methodological limitation contributes to inconsistencies in the identification and interpretation of determinants across studies.

All these limitations suggest that food and nutritional insecurity in older adults remains insufficiently understood as a multidimensional and interconnected phenomenon. Although a wide range of determinants has been identified, existing studies have largely examined them in isolation, limiting a comprehensive understanding of how these factors interact and reinforce vulnerability over time.

Given the rapid evolution of social inequalities, demographic aging, and food systems in recent years, there is a need to focus on more contemporary evidence that reflects current contexts and incorporates a broader, multidimensional perspective. Therefore, this review prioritizes studies published from 2015 onwards to ensure the inclusion of more recent, methodologically comparable, and contextually relevant evidence.

In this context, this scoping review aims to map and synthesize the available scientific evidence on the main factors associated with FNI in this population, considering socioeconomic, health, functional, psychosocial and structural dimensions. By providing a comprehensive and structured overview of these determinants, this study seeks to support the development of more effective interventions and inform intersectoral policies aimed at promoting food security and healthy, dignified aging.

## 2. Materials and Methods

A scoping review was conducted given the heterogeneity of the evidence and the need to map the range and interactions of FNI. The study follows the Joanna Briggs Institute (JBI) Manual for Evidence Synthesis [12], and the Preferred Reporting Items for Systematic Reviews and Meta-Analyses extension for Scoping Reviews (PRISMA-ScR) [13,14] for the reporting. The protocol was registered on FIGSHARE (10.6084/m9.figshare.29356109), and the review question “What are the determining factors of food and nutritional insecurity in older adults?” was defined based on the Population–Concept–Context (PCC) framework as follows in Table 1.

### 2.1. Search Strategy

A comprehensive search was conducted on 24 November 2024, using the following electronic, international and public databases: PubMed/MEDLINE, EMBASE, Scopus, Web of Science, CINAHL, LILACS, ProQuest and Google Scholar. The descriptors were selected from Medical Subject Headings (MeSH) and Descriptors in Health Sciences (DeCS). A reverse manual search was also performed to identify relevant articles cited in all selected studies. The database search strategies (available in Supplementary Table S1), were developed with the assistance of a librarian, applying specific combinations of descriptors, Boolean operators and truncations to ensure comprehensiveness and precision of the results.

### 2.2. Eligibility Criteria

All studies addressing aspects of food insecurity in affected older adults (≥60 years old), as well as those referencing the determining factors that lead to food insecurity in this population, were considered. Non-original studies (commentaries, reviews, letters to the editor, case reports, theses, and abstracts) and those that did not specify the dimensions of the FNI experience or did not include older adults in that context were excluded. Studies published before 2015 were considered ineligible to ensure that the analysis reflects more recent evidence, considering advances in measurement instruments, as well as changes in socioeconomic conditions and public policies related to food insecurity in older adults.

### 2.3. Study Selection

Study selection was conducted independently by two reviewers in three stages: title analysis, abstract reading and full-text reading and comprehension. Regular calibration meetings were held to ensure alignment between reviewers. Disagreements were resolved through discussion and consensus, with the involvement of a third reviewer when necessary. Duplicate records were identified and removed using EndNote^®^ (basic version, 2025) and the screening process was conducted via Rayyan QCRY^®^ Software (free web version), which ensured blinded and independent assessment among the authors.

A detailed record of exclusion reasons was maintained during the full-text review stage to ensure transparency.

### 2.4. Data Extraction

A data extraction table tool was developed in Microsoft Office^®^ Excel independently by the researchers, to extract the primary relevant information. This process was performed individually by each author, and results were compared to address discrepancies and improve accuracy. After the screening process, the results were compiled including the evidence extracted separately by each author and subsequently approved by all. The extracted data included: authors, country, year of publication, study design, assessment tools, determinants of FNI, and key findings.

## 3. Results

**Figure 1:**
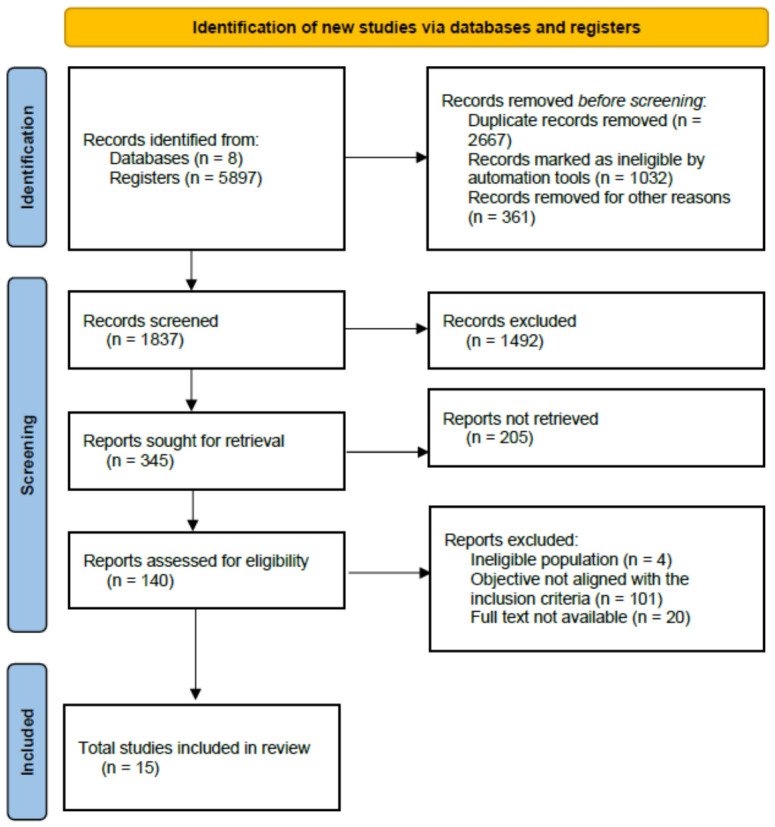
PRISMA flow diagram.

A total of 5897 records were identified through electronic database searches. Following the removal of duplicates, 1837 studies underwent title and abstract screening, leading to the retrieval of 140 articles for full-text eligibility assessment. The application of the eligibility criteria, combined with the limited number of studies explicitly addressing the multidimensional determinants of FNI in older adults, resulted in a focused selection. Ultimately, 15 studies fulfilled all inclusion criteria and were incorporated into this scoping review (Figure 1).

### 3.1. Characteristics of Selected Studies

The main characteristics of the 15 included studies are summarized in Table 2. Most of the included studies were conducted in North America, particularly in the United States (*n* = 6; 40%) and Canada (*n* = 2; 13.3%). The remaining studies were conducted in Europe (Portugal, *n* = 2), Latin America (Brazil and Colombia, *n* = 2), and Asia and Africa (Nepal, Ghana, and India, *n* = 3). Regarding study design, the majority were cross-sectional studies (*n* = 13) [8,15,16,17,18,19,20,21,22,23,24,25,26], along with one cohort study [27] and one mixed-methods study [28] (Table 2).

### 3.2. Assessment Tools

FNI was primarily assessed using validated psychometric instruments (*n* = 10), including the Food Insecurity Experience Scale (FIES), the U.S. Household Food Security Survey Module (HFSSM), the Latin American and Caribbean Food Security Scale (ELCSA), and the Brazilian Food Insecurity Scale (EBIA). The HFSSM was the most frequently used tool.

Five studies assessed FNI using alternative approaches, including large-scale surveys such as the Canadian Community Health Survey (CCHS) and the World Health Organization (WHO) Study on Global Ageing and Adult Health (SAGE), as well as screening tools and proxy indicators derived from broader datasets such as the Health and Retirement Study (HRS).

Only two studies incorporated specific tools to assess malnutrition, namely the Mini Nutritional Assessment (MNA), sometimes complemented by anthropometric measurements. Additionally, some studies evaluated psychosocial dimensions using instruments such as the Multidimensional Scale of Perceived Social Support (MSPSS), the Gijón Social–Family Evaluation Scale, and the WHO Quality of Life (WHOQoL) scale. Finally, one study explored biological dimensions by examining epigenetic aging through Venous Blood Study (VBS) samples, highlighting emerging approaches in the assessment of food insecurity (Table 2).

### 3.3. Determinants of Food and Nutrition Insecurity

The included studies identified a wide range of determinants of FNI among older adults, which were grouped into five primary domains: socioeconomic, health-related, functional, psychosocial, and structural. Across the literature, socioeconomic determinants were the most consistently reported (*n* = 15), followed by health-related (*n* = 13), psychosocial (*n* = 12), structural (*n* = 11), and functional factors (*n* = 9). The frequent identification of multiple coexisting factors reinforces the inherently multidimensional nature of FNI.

Regarding the direction of association, lower income, limited educational attainment, and financial strain were consistently linked to an increased risk of FNI, suggesting that economic vulnerability remains the central underlying driver. Similarly, poor health status, multimorbidity, and depressive symptoms showed robust associations across studies, indicating a bidirectional relationship between health and nutritional security.

Psychosocial factors, particularly loneliness and limited social support, were also frequently associated with FNI, although their impact appears to be context-dependent and often mediated by socioeconomic conditions. At the structural level, barriers to accessing food assistance and systemic inequalities further compound these vulnerabilities, highlighting the critical role of broader contextual factors.

Overall, these findings suggest that FNI in older adults is best understood as a result of interacting and cumulative vulnerabilities, with socioeconomic disadvantage acting as the central axis through which other determinants operate.

#### 3.3.1. Socioeconomic Determinants

Socioeconomic factors emerged as the most consistent and influential determinants of food insecurity among older adults. Economic vulnerability, particularly low income, financial instability, and poverty, was strongly and consistently associated with increased risk across studies [8,15,16,17,18,19,21,22,24,25,26,27,28], suggesting that financial constraints represent the primary underlying driver of food insecurity.

Additional factors, including lower educational attainment, reduced wealth, and unemployment further compounded this vulnerability [22,25,27]. Living arrangements and social position also played a role, with individuals living alone, being unmarried, or belonging to minority groups facing higher risk [8,18,26]. Subjective financial strain reinforced these patterns, highlighting the importance of perceived as well as objective economic hardship [17]. Conversely, stable income sources, pensions, remittances, and ownership of productive assets appeared to mitigate risk, underscoring the protective role of financial security in later life [18].

#### 3.3.2. Health-Related Determinants

Health-related factors demonstrated a bidirectional relationship with food insecurity. Chronic diseases, multimorbidity, and poor self-rated health were consistently associated with increased vulnerability [16,22,24,26,28], while food insecurity itself was linked to adverse outcomes, including depression, poorer quality of life, and deteriorating physical and mental health [19,26].

These findings suggest a reinforcing cycle in which health and food insecurity mutually exacerbate each other. Nutritional status was also affected, with evidence of both undernutrition and higher BMI among food-insecure individuals [15,24], reflecting the complex nutritional implications of food insecurity in older populations.

Additionally, behavioral and biological factors, such as smoking and accelerated epigenetic aging were associated with FNI [18,25], indicating that its impact extends beyond immediate nutritional outcomes. Some variables, including participation in social assistance programs and marital status appeared to reflect underlying vulnerability rather than acting as direct determinants [21].

#### 3.3.3. Functional Determinants

Functional limitations were consistently associated with increased vulnerability to food insecurity among older adults. Reduced mobility, frailty, and difficulties in performing activities of daily living limit the ability to acquire, prepare, and consume food, thereby compromising autonomy and access [16,19,23,26].

These findings suggest that functional decline acts as both a direct barrier to food access and an indirect contributor through reduced independence. Lower levels of physical activity were also associated with FNI [25], reinforcing the interrelationship between functional capacity and nutritional vulnerability.

#### 3.3.4. Psychosocial Determinants

Psychosocial factors particularly loneliness, social isolation, and limited social support were consistently associated with increased risk of FNI [8,15,16,21,23,26,28]. These factors appear to influence food access both directly, by reducing practical support, and indirectly, through their impact on mental health and well-being.

Living alone and weak social networks further intensified vulnerability, whereas strong social support acted as a protective factor [8]. Depressive symptoms, psychological stress, and perceived economic insufficiency also contributed to FNI [17,25], highlighting the complex interplay between emotional, social, and economic dimensions.

Additionally, household dynamics and cultural norms influenced food distribution and access within families [18], indicating that psychosocial determinants operate within broader social contexts.

#### 3.3.5. Structural Determinants

Structural determinants reflect the broader environmental and policy contexts shaping FNI. Barriers related to transportation, geographic inequalities, and restricted access to food assistance programs were consistently identified as key contributors [16,23,28]. These factors are embedded within wider socioeconomic inequalities, including disparities in healthcare access and social protection systems, which further exacerbate vulnerability [19,27]. While participation in social programs and community services was associated with reduced food insecurity, their impact was often limited, suggesting gaps in coverage and accessibility [15,20]. Regional disparities, rural underdevelopment, and structural inequalities affecting minority populations further intensified risk [8,18,22,26]. The low participation in assistance programs highlights persistent structural barriers [25], indicating that availability alone is insufficient without equitable access.

## 4. Discussion

This review contributes to the existing literature by providing an integrated and multidimensional synthesis of FNI among older adults. Rather than introducing new categories, the added value of this study lies in conceptualizing FNI as a system of interacting and cumulative vulnerabilities, in which socioeconomic, health-related, functional, psychosocial, and structural factors do not operate independently but reinforce each other over time. These findings are consistent with broader literature on food insecurity and social determinants of health, which emphasizes the central role of socioeconomic inequality, health status, and social vulnerability in shaping access to adequate nutrition in later life.

Socioeconomic disadvantage emerged as the most consistent determinant, with low income, limited educational attainment and financial instability [16,18], consistently linked to higher risk across various geographical contexts [19,20]. Such conditions constrain food access and diminish the capacity of older adults to manage age-related challenges including rising healthcare costs and post-retirement income shifts [23,26]. Additionally, subjective perceptions of income insufficiency appear to exacerbate these vulnerabilities [14], suggesting that perceived economic strain is as critical as absolute poverty.

Health-related and functional determinants also play a central role. Chronic diseases and multimorbidity often coexist with functional limitations, impairing the ability to acquire and prepare food while simultaneously increasing nutritional demands [25,27]. This creates a detrimental cycle where poor health and FNI mutually reinforce physiological decline.

Psychosocial factors, particularly social isolation, widowhood, and limited support networks, further compound this risk [1,20,24,27]. The absence of social support not only restricts physical access to food but also negatively affects emotional well-being and dietary behaviors [22].

At the structural level, this review identifies systemic barriers, including institutional inequalities and exclusionary policies, as key contributors to FNI, particularly among marginalized populations [25,27]. Evidence from different contexts shows that vulnerable groups often experience higher levels of food insecurity, even when controlling for economic factors, highlighting that FNI is deeply embedded in broader systems of inequality. Additionally, gender disparities are evident, with older women showing greater vulnerability [16,21], likely reflecting cumulative disadvantages in access to resources over the life course.

### 4.1. Measurement Challenges and Implications

The findings reveal substantial heterogeneity in the measurement of FNI across studies [15,16,17,18,19,20,21,22,25,27,28]. Although validated instruments such as the FIES, the HFSSM, the ELCSA, and the EBIA are widely used, differences in conceptualization and application persist.

While these tools effectively capture economic aspects of food access, they may not fully reflect dimensions particularly relevant to older adults, including functional limitations, psychosocial vulnerability, and health-related constraints [25,27]. Simplified screening instruments, although useful in clinical practice, may underestimate the complexity of FNI in this population [1].

This lack of standardization limits comparability across studies and may hinder the accurate identification of high-risk groups. These findings highlight the need for more comprehensive, multidimensional and context-sensitive measurement approaches that better capture the complexity of FNI in older populations.

### 4.2. Implications for Policy and Practice

The evidence synthesized in this review suggests the importance of integrated, intersectoral responses to address FNI among older adults [20,22,25,27]. Policies may benefit from strengthening social protection systems and reducing barriers to accessing food assistance programs. Moreover, interventions that combine health and social care, such as nutritional support, chronic disease management, and strategies to reduce social isolation, may help mitigate vulnerability in this population [17,24]. Community-based approaches that strengthen social networks could play a particularly important role, especially for vulnerable subgroups such as older women and individuals living alone [16,21,25].

Given the heterogeneity and limited number of studies included, these implications should be interpreted with caution. However, they provide a useful basis for informing future policy development and practice.

### 4.3. Limitations

Several limitations should be considered. The heterogeneity of study designs and measurement tools may limit comparability across findings. The exclusion of studies published before 2015 may have resulted in the omission of earlier relevant evidence. Although no language restrictions were applied, potential language bias cannot be entirely excluded due to database indexing limitations. Finally, as a scoping review, this study does not include a formal assessment of methodological quality, which should be considered when interpreting the findings.

## 5. Conclusions

Food and nutrition insecurity in older adults is a complex and multidimensional phenomenon shaped by the interaction of social, economic, health, and structural determinants. Rather than resulting from isolated risk factors, it reflects the cumulative effect of overlapping vulnerabilities that evolve across the ageing process.

Addressing FNI in later life requires moving beyond fragmented interventions toward integrated, multisectoral strategies. Policy responses should prioritize income security, access to healthcare, and the strengthening of social support networks, while also addressing structural inequalities that disproportionately affect vulnerable groups. Such equity-oriented approaches are essential to improve food access and support healthy and dignified ageing.

By synthesizing recent evidence, this study offers an integrative perspective on the determinants of FNI and highlights the need for future research to develop models that capture their interactions, as well as to advance the standardization of measurement approaches to improve comparability across contexts.

## Figures and Tables

**Table 1:** PCC framework.

Population	Concept	Context
Older adults	Food Insecurity	Healthcare
Aged; Seniors	Food shortage	
Elderly	Food Ration	
Centenarian	Determinants	
Geriatrics	Social Determinants	

**Table 2:** Characteristics of selected studies.

Author/Year/Country	Study Design	Assessment Tools	Determinants of FNI *	Key Findings
Kansanga et al., 2022 Canada [8]	Cross-Sectional	CCHS *, HRS *	Socioeconomic, Health-Related, Psychosocial and Structural	Higher FNI risk was significantly associated with: **Minority groups** (OR = 1.29, *p* < 0.01); **Living alone** (OR = 1.13, *p* < 0.05); **Weak community belonging** (OR = 1.40, *p* < 0.001); **Poor physical health** (OR = 1.20, *p* < 0.01). Lower age brackets and lower income levels were also identified as significant predictors.
Ganhão et al., 2018 Portugal [15]	Cross-Sectional	FIES *, GSFES *, MNA *	Socioeconomic, Health-Related, Psychosocial and Structural,	High FNI prevalence (70.0%). 40.7% of participants were at risk of malnutrition and 4.7% were malnourished. Over one-third (34.7%) presented elevated social risk.
Kihlström et al., 2018 USA [16]	Cross-Sectional	HFSSM *, MSPSS *	Association between food insecurity and health-related quality of life.	Food-insecure individuals were significantly more likely to report ≥14 days with activity limitations (OR = 4.07, 95% CI 0.68–24.1) and ≥14 physically unhealthy days (OR = 1.49). FNI identified as a critical social determinant of health.
Maia et al., 2019 Portugal [17]	Cross-Sectional	HFSSM *	Socioeconomic, Psychosocial,	FNI (16.6% prevalence) was strongly associated with: Perceived income insufficiency (OR = 10.39); Low education (OR = 5.46); Female sex (OR = 1.96); Unmarried status (OR = 1.79); Lower-level occupations (OR = 2.22).
Singh et al., 2020 Nepal [18]	Cross-Sectional	HFSSM *	Socioeconomic, Health-Related, Psychosocial and Structural	41.1% FNI prevalence. Risk doubled for families with monthly income <$100 (AOR = 2.26, 95% CI 1.08–4.76). Conversely, adult children migration acted as a protective factor (AOR = 0.47).
Estrada-Restrepo et al., 2022 Colombia [19]	Observational Cross-Sectional	ELCSA *	Socioeconomic, Health-Related, Functional, Psychosocial and Structural	55% FNI prevalence. Risk significantly higher in rural settings and larger households (*p* < 0.05). Protective factors included households consisting only of older adults (OR = 0.58) or led by individuals ≥60 years (OR = 0.64).
Queiroz et al., 2022 Brazil [20]	Cross-Sectional	EBIA *, MNA *	Socioeconomic, Health-Related and Structural	63.5% FNI prevalence; 35.2% were malnourished or at risk. Moderate to severe FNI increased the likelihood of malnutrition nearly three-fold (OR = 2.97, 95% CI 1.37–6.44).
Burris et al., 2019 USA [21]	Cross-Sectional	HFSSM *	Socioeconomic, Health-Related and Psychosocial	Significant risk factors for FNI included: **Loneliness** (OR = 1.356, *p* = 0.005); **Divorced status** (OR = 0.208, *p* = 0.008); * **SNAP participation** (OR = 4.765, *p* = 0.003). Limited social support was also a key predictor (*p* = 0.001).
Leung et al., 2020 USA [22]	Cross-Sectional	HFSSM *	Socioeconomic, Health-Related and Structural	FNI (14% prevalence) was strongly associated with multimorbidity: **4–10 chronic conditions** (RRR = 2.59) vs. **2–3 conditions** (RRR = 1.60). Fair/poor self-rated health increased risk five-fold (RRR = 5.13).
Tetteh et al., 2024 Ghana [23]	Cross-Sectional	HFSSM *, SAGE *	Socioeconomic, Health-Related, Functional, Psychosocial and Structural	28% FNI prevalence. Linked to adverse outcomes: **Depression** (PR = 3.43); **Quality of life decline** (PR = 2.01); **Functional disability** (PR = 1.18); **Declining memory performance** (aβ = −0.85).
Pirrie et al., 2020 Canada [24]	Cross-Sectional	BHST *	Socioeconomic, Health-Related and Psychosocial	FNI was strongly associated with: **Poverty** (AOR = 23.52, 95% CI 8.75–63.22); **Being underweight** (AOR = 19.79, 95% CI 1.91–204.80). Anxiety/depression also significantly increased vulnerability.
Tamargo et al., 2024 USA [25]	Cross-Sectional	HFSSM *, VBS *	Socioeconomic, Health-Related, Functional, Psychosocial and Structural	FNI (8.1% prevalence) was linked to accelerated **epigenetic aging**, specifically markers Zhang (B = 0.09, *p* = 0.011) and GrimAge (B = 0.57, *p* = 0.022). Risk factors included minority status, low wealth, and high * BMI.
Selvamani et al., 2023 India [26]	Cross-Sectional	SAGE *, WHOQoL *	Socioeconomic, Health-Related, Functional, Psychosocial and Structural	Severe FNI doubled the likelihood of **poor quality of life** (OR = 2.49) and **low life satisfaction** (OR = 2.36). Associated with a 3.60-point reduction in WHO-QoL scores (*p* < 0.001).
Leung et al., 2024 USA [27]	Cohort	HFSSM *, MSPSS *	Socioeconomic, Health-Related and Structural	U.S. household FNI rose from 12.5% to 23.1% (1999–2019). Chronic FNI tripled (2.0% to 6.3%). Higher prevalence consistently observed among Black and Hispanic households and those with lower socioeconomic status.
Aday et al., 2022 USA [28]	Mixed-Methods	HFSSM *	Socioeconomic, Health-Related, Functional, Psychosocial and Structural	30% presented FNI (marginal to very low). Vulnerability peaked among older women, widowed/divorced individuals, and those below the poverty threshold. Key barriers: financial constraints, transportation, and health limitations.

* BMI: Body Mass Index, * BHST: Brief Hunger Screening Tool, * EBIA: Brazilian Food Insecurity Scale, * ELCSA: Latin American and Caribbean Food Security Scale, * CCHS: Canadian Community Health Survey, * FD: Functional Difficulties, * FIES: Food Insecurity Experience Scale, * GSFES: Gijón Social–Family Evaluation Scale, * HAQ: Health Assessment Questionnaire, * HRS: Health and Retirement Study, * Qol: low Quality of Life, * MNA: Mini Nutritional Assessment, * MSPSS: Multidimensional Scale of Perceived Social Support, * SNAP: Supplemental Nutrition Assistance Program, * SAGE: Study on Global Ageing and Adult Health, * HFSSM: U.S. Household Food Security Survey Module, * UNHS: Unmet Needs of Health Services, * VBS: Venous Blood Study, * WHOQoL: WHO Quality of Life Scale.

## Data Availability

No new data were created or analyzed in this study. Data sharing is not applicable to this article.

## Supplementary Materials

The following supporting information can be downloaded at: https://www.mdpi.com/article/10.3390/healthcare14101396/s1, Table S1: Search strategies to the different databases.

## Abbreviations

The following abbreviations are used in this manuscript:

BMI	Body Mass Index
EBIA	Brazilian Food Insecurity Scale
BHST	Brief Hunger Screening Tool
CCHS	Canadian Community Health Survey
DeCS	Descriptors in Health Sciences
FNI	Food and nutritional insecurity
FI	Food insecurity
FIES	Food Insecurity Experience Scale
FD	Functional Difficulties
GSFES	Gijón Social–Family Evaluation Scale
HRS	Health and Retirement Study
HFSSM	Household Food Security Survey Module
JBI	Joanna Briggs Institute
ELCSA	Latin American and Caribbean Food Security Scale
MeSH	Medical Subject Headings
MNA	Mini Nutritional Assessment
MSPSS	Multidimensional Scale of Perceived Social Support
PCC	Population–Concept–Context
PRISMA-ScR	Preferred Reporting Items for Systematic Reviews and Meta-Analyses extension for Scoping Reviews
Qol	Quality of Life
SAGE	Study on Global Ageing and Adult Health
SNAP	Supplemental Nutrition Assistance Program
UNHS	Unmet Needs of Health Services
VBS	Venous Blood Study
WHOQoL	WHO Quality of Life Scale
