# Supplementary material for: Multidimensional Determinants of Food and Nutritional Insecurity Among Older Adults: A Scoping Review
Source: Healthcare (Basel). 2026 May 20;14(10):1396. doi: 10.3390/healthcare14101396 (PMC13205513; doi:10.3390/healthcare14101396)
Supplement: Supplementary file 1 [file healthcare-14-01396-s001.zip › healthcare-4245229-supplementary.pdf]

**Table S1.** Search Strategies to Different Databases.

| Database | Search Strategy | Results |
|---|---|---|
| Medline / PubMed | ("Food Insecurity"[MeSH Terms] OR "food insecurit*"[Title/Abstract] OR "food ration*"[Title/Abstract] OR "food shortage*"[Title/Abstract]) AND ("Social Determinants of Health"[MeSH Terms] OR "determin*"[Title/Abstract] OR "SDH"[Title/Abstract]) AND ("Aged"[MeSH Terms] OR "Aged"[Title/Abstract] OR "Elderly"[Title/Abstract] OR "older adult*"[Title/Abstract] OR "aged, 80 and over"[MeSH Terms] OR "80 and over"[Title/Abstract] OR "Oldest Old"[Title/Abstract] OR "nonagenarian*"[Title/Abstract] OR "octogenarian*"[Title/Abstract] OR "centenarian*"[Title/Abstract] OR "Geriatrics"[MeSH Terms] OR "geriatric*"[Title/Abstract] OR "senior*"[Title/Abstract] OR "senium"[Title/Abstract] OR "Middle Aged"[MeSH Terms] OR "middle age*"[Title/Abstract]) | 974 |
| Embase | ('food insecurity'/exp OR 'food insecurit*':ti,ab,kw OR 'food ration*':ti,ab,kw OR 'food shortage*':ti,ab,kw OR 'food shortage'/exp) AND ('social determinants of health'/exp OR 'determin*':ti,ab,kw OR 'sdh':ti,ab,kw) AND ('aged'/exp OR 'aged':ti,ab,kw OR 'elderly':ti,ab,kw OR 'older adult*':ti,ab,kw OR 'very elderly'/exp OR '80 and over':ti,ab,kw OR 'oldest old':ti,ab,kw OR 'nonagenarian*':ti,ab,kw OR 'octogenarian*':ti,ab,kw OR 'centenarian*':ti,ab,kw OR 'geriatrics'/exp OR 'geriatric*':ti,ab,kw OR 'senior*':ti,ab,kw OR 'senium':ti,ab,kw OR 'middle aged'/exp OR 'middle age*':ti,ab,kw) | **1,357** |
| Scopus | (TITLE-ABS-KEY("Food Insecurit*" OR "Food Ration*" OR "food shortage*")) AND (TITLE-ABS-KEY(Determin* OR "SDH")) AND (TITLE-ABS-KEY(Aged OR Elderly OR "Older adult*" OR "80 and over" OR "Oldest Old" OR Nonagenarian* OR Octogenarian* OR Centenarian* OR geriatric* OR senior* OR senium OR "Middle Age*")) | **1,387** |
| Web of Science | TS=("Food Insecurit*" OR "Food Ration*" OR "food shortage*") AND TS=(Determin* OR "SDH") AND TS=(Aged OR Elderly OR "Older adult*" OR "80 and over" OR "Oldest Old" OR Nonagenarian* OR Octogenarian* OR Centenarian* OR geriatric* OR senior* OR senium OR "Middle Age*") | **1,567** |
| CINAHL | TI ( ("Food Insecurit*" OR "Food Ration*" OR "food shortage*") AND (Determin* OR "SDH") AND (Aged OR Elderly OR "Older adult*" OR "80 and over" OR "Oldest Old" OR Nonagenarian* OR Octogenarian* OR Centenarian* OR geriatric* OR senior* OR senium OR "Middle Age*") ) OR AB ( ("Food Insecurit*" OR "Food Ration*" OR "food shortage*") AND (Determin* OR "SDH") AND (Aged OR Elderly OR "Older adult*" OR "80 and over" OR "Oldest Old" OR Nonagenarian* OR Octogenarian* OR Centenarian* OR geriatric* OR senior* OR senium OR "Middle Age*") ) OR SU ( ("Food Insecurit*" OR "Food Ration*" OR "food shortage*") AND (Determin* OR "SDH") AND (Aged OR Elderly OR "Older adult*" OR "80 and over" OR "Oldest Old" OR Nonagenarian* OR Octogenarian* OR Centenarian* OR geriatric* OR senior* OR senium OR "Middle Age*") ) | **234** |
| LILACS | ("Food Insecurity" OR "Food Insecurities" OR "food shortage" OR "food shortages" OR "Food Rationing" OR "Insegurança Alimentar" OR "Racionamento de Alimentos" OR "Racionamento de Comida" OR "Inseguridad Alimentaria" OR "Racionamiento Alimentario" OR "racionamiento de los alimentos") AND (determinant OR determinants OR "SDH" OR determinante OR determinantes ) AND (aged OR elderly OR "Older adult" OR "Older adults" OR "80 and over" OR "Oldest Old" OR nonagenarian OR octogenarian OR centenarian OR geriatric OR senior OR senium OR "Middle Aged" OR nonagenarians OR octogenarians OR centenarians OR geriatrics OR idoso OR idosos OR idosa OR idosas OR "Pessoa de Idade" OR "Pessoas de Idade" OR anciano OR ancianos OR "Adulto Mayor" OR "Persona Mayor" OR "Persona de Edad" OR "Personas Mayores" OR "Personas de Edad" OR "Idoso de 80 Anos ou mais" OR centenarios OR nonagenarios OR octogenarios OR velhíssimos OR "Anciano de 80 o más Años" OR viejísimos OR geriátrico OR geriátricos OR geriátrica OR geriátricas OR "Meia Idade" OR "Mediana Edad" ) AND db:("LILACS") AND instance:"regional" | **23** |
| ProQuest | TS=("Food Insecurit*" OR "Food Ration*" OR "food shortage*") AND TS=(Determin* OR "SDH") AND TS=(Aged OR Elderly OR "Older adult*" OR "80 and over" OR "Oldest Old" OR | **255** |

| | | |
|---|---|---|
| | Nonagenarian* OR Octogenarian* OR Centenarian* OR geriatric* OR senior* OR senium OR "Middle Age*") | |
| Google Scholar | ("Food Insecurity" OR "Food Insecurities" OR "food shortage" OR "food shortages" OR "Food Rationing" OR "Insegurança Alimentar" OR "Racionamento de Alimentos" OR "Racionamento de Comida" OR "Inseguridad Alimentaria" OR "Racionamiento Alimentario" OR "racionamiento de los alimentos") AND (Determinant OR Determinants OR "SDH" OR Determinante OR Determinantes) AND (Aged OR Elderly OR "Older adult" OR "Older adults" OR geriatric OR senior OR geriatrics OR Idoso OR Idosos OR Idosa OR Idosas OR "Pessoa de Idade" OR "Pessoas de Idade" OR Anciano OR Ancianos OR "Adulto Mayor" OR "Persona Mayor" OR "Persona de Edad" OR "Personas Mayores" OR "Personas de Edad" OR Velhíssimos OR Viejísimos OR geriátrico OR geriátricos OR geriátrica OR geriátricas) | **100** |
